# Morphological and pathological response in primary systemic therapy of patients with breast cancer and the prediction of disease free survival: a single center observational study

**DOI:** 10.3325/cmj.2016.57.131

**Published:** 2016-04

**Authors:** Gyöngyvér Szentmártoni, Anna-Mária Tőkés, Timea Tőkés, Krisztián Somlai, Attila Marcell Szász, László Torgyík, Janina Kulka, Magdolna Dank

**Affiliations:** 1 Semmelweis University, Department of Clinical Oncology, Budapest, Hungary; 2 2nd Department of Pathology, Budapest, Hungary; 3 Surgical Division of St. Margit Hospital, Budapest, Hungary

## Abstract

**Aim:**

To identify breast cancer subtypes likely to respond to primary systemic therapy (PST or neoadjuvant therapy) and to assess the accuracy of physical examination (PE) and breast ultrasonography (US) in evaluating and predicting residual size of breast carcinoma following PST.

**Methods:**

116 patients who received at least two cycles of PST between 1998 and 2009 were selected from a prospectively collected clinical database. Radiological assessment was done by mammography and US. Prior to PST, tumors were subclassified according to core biopsy (NCB) and/or fine-needle aspiration-based immunohistochemical profiles of NCB. Pathological response rates were assessed following the surgeries by using Chevallier classification. Tumor measurements by PE and US were obtained before and after PST. Different clinical measurements were compared with histological findings. Disease-free survival (DFS) was assessed.

**Results:**

Pathological complete remission (pCR = Chevallier I/II) was observed in 25 patients (21.5%), 44% of whom had triple negative histology, 28% Her2 positive and 76% had high-grade tumor. Of 116 patients, 24 received taxane-based PST, 48 combined taxane + anthracycline treatment, 8 trastuzumab combinations, 21 anthracycline-based treatments, and 15 other treatments. In the taxane treated group, the pCR rate was 30%, in the taxane + anthracycline group 25%, in the anthracycline group 9.5%, and in trastuzumab group 37.5%. After PST, PE and US were both significantly associated with pathology (*P* < 0.001 and *P* = 0.004, respectively). Concerning OS, significant difference was observed between the Chevallier III and IV group (*P* = 0.031) in favor of Chevallier III group. In the pCR group, fewer events were observed during the follow-up period.

**Conclusions:**

Our results show that even limited, routinely used immunohistochemical profiling of tumors can predict the likelihood of pCR to PST: patients with triple negative and Her2-positive cancers are more likely to achieve pCR to PST. Also, PE is better correlated with pathological findings than US.

There are many controversial data on the benefits and risks of primary systemic therapy (PST) of breast cancer. It is generally accepted that PST results in various clinical responses in 60%-90% of patients, while pathologic complete remission (pCR), the predictor of overall survival, occurs only in 3%-16% of patients (1-3).

Although the response rate of breast cancers to PST is a short-term marker, it has a long-term outcome and important influence on patients’ life. Therefore, it is important to identify new and reliable factors that may predict response to PST. Several studies have been conducted with the aim to identify predictive factors for pCR after administration of PST. Early identification of features that can predict pCR may allow a better selection of patients. However, there is no global consensus on the predictive factors. The role of hormone receptor status, tumor grade, and tumor cell proliferation has already been established (1-8). A large number of studies showed that women with luminal A type cancer (ER-positive/Her2-negative) were unlikely to achieve a pCR after optimal neoadjuvant chemotherapy (9,10). Based on this observation, some experts consider that patients with luminal A tumors are not eligible for preoperative chemotherapy (11).

Controversies exist in the assessment of the accuracy of physical examination, sonography, and mammography in predicting the residual size of breast tumors following PST (12,13). Physical examination is one of the accepted clinical standards in the evaluation of tumor size before, during, and after neoadjuvant chemotherapy, while pathological evaluation is the gold standard and the ultimate assessment modality of the residual tumor size after neoadjuvant chemotherapy (14,15). Ultrasound (US) is used primarily for diagnostic purposes – size and biopsy – and for wire localization. It is considered complementary to mammography and PE. The sensitivity and specificity of PE and US vary in different studies (1,12,16,17). Sperber et al correlated the findings of PE, US and mammography performed by the same oncologic and radiologic team in patients with locally advanced breast cancer or a tumor/breast tissue ratio that precludes breast-conserving surgery. They found that none of these methods adequately delineated the real extent of the disease in the breast and axillary lymph nodes (18). Peintinger et al calculated the agreement between the predicted and the pathologic responses and the predicted and pathologic tumor sizes by using PE, mammography, and US at diagnosis and before surgery in 162 breast cancer patients who received PST. They found that the overall agreement between predicted and pathologic responses was 53% for PE, 67% for mammography plus US, and 63% for PE plus mammography and US. The sensitivity of mammography and US in predicting pCR was 78.6%, the specificity was 92.5%, and the accuracy was 88.9. Agreement of residual tumor size in mammography and US with pathologic residual tumor size was moderate (18,19). In a recent study, the US estimated pathological tumor size correctly in 63%, overestimated it in 20%, and underestimated it in 17% of 182 patients who underwent PST. However, US was as least as good as breast MRI (20).

Given the important role of the assessment of residual tumor size in determining the surgical procedure after neoadjuvant chemotherapy the aims of this study were:

1) to prospectively evaluate the accuracy of PE and US for clinical staging of primary breast cancer in women receiving neoadjuvant chemotherapy. Until rebiopsy after first cycle of therapy or novel molecular imaging methods will be available in the everyday practice, we need to establish which of the conventional evaluating methods has the highest predictive value for pCR;

2) to compare the results with pathologic measurement performed on surgical specimens;

3) to determine the breast cancer subgroups likely to respond to neoadjuvant chemotherapy;

4) to correlate the results of pathological response evaluation (ie, Chevallier classification) with the disease free survival (DFS).

## Methods

### Patients

116 patients who received at least two cycles of PST between 1998 and 2009 were selected from a prospectively collected clinical database. Patients were not included in the study if there was evidence of inflammatory breast cancer, metastatic disease, previous hormonal therapy for breast cancer, and surgery and radiotherapy. Radiological assessment was done by mammography and US (PET/CT and MRI were only available in the second part of the analyzed period therefore not considered in this study). Tumor measurements by PE, and US were performed before and after PST. Prior to PST, tumors were subclassified according to core biopsy (NCB) and/or fine-needle aspiration-based immunohistochemical profiles of NCB. Pathological response rates were assessed following the surgeries by using Chevallier classification. Different clinical measurements were compared with histological findings. Disease-free survival was assessed. Distant metastases were screened by chest x-ray, abdominal sonography, or by CT scan/PET CT.

### Clinical assessment

Clinical measurements (physical examination and/or breast sonography) were performed before treatment, at every two or three cycles during therapy, and at the end of neoadjuvant treatment. The average number of treatment cycles was 5.6; the majority of patients underwent 6 cycles.

Data on the PE of the tumors were available in 108/116 patients and on US in 58/116 patients. Clinical palpation, ultrasonography, and treatment were performed by the same well trained team consisting of oncologists and radiologists at Semmelweis University. Regarding PE, palpation and caliper measurements were performed by the treating physician. Breast US was routinely performed before and after PST by the same experienced radiologist in our institute. Those US results that did not meet these criteria were excluded from the analysis. Patients’ demographics, tumor characteristics, and the largest diameter of the multidimensional tumor measurement obtained by physical examination and/or sonography were recorded. The findings were compared with pathological staging.

The clinical response to neoadjuvant chemotherapy was classified according to the Union for International Cancer Control (UICC) criteria (cCR – complete response; cPR – partial response; cSD – stable disease; and cPD – progressive disease) (21,22).

### Pathological assessment

Histopathological diagnosis, hormone receptor status, and Her2/neu status were determined based on the core biopsy or fine-needle aspiration biopsy (FNAB) before neoadjuvant therapy. Estrogen (ER) and progesterone receptor (PR) status were determined by using 6F11 and the PR clone 312 (both from Novocastra Laboratories Ltd, Burlingame, CA, USA), respectively, and standard immunohistochemical methods. Tumors with >10% stained cells were considered to have positive receptor status. HER2/neu status was assessed by immunohistochemistry (HER2/neu CB11, Novocastra Laboratories Ltd). CB11 was scored by experienced pathologists according to approved guidelines (23). In 23 cases, the fluorescence in situ hybridization (FISH) data were available. FISH was performed again in this cases by using a fluorescein-labeled HER2 probe (ERBB2, Her2/Neu, Kreatech Diagnostics, Amsterdam, The Netherlands) and automated technique (Ventana Medical Systems, Inc., Tucson, AZ, USA) (24-26).

Sections analyzed by FISH were adjacent to the section used for immunohistochemistry and the same areas of the tumors were evaluated.

Biological subtypes of tumors were defined according to the recommendations of the 13th St. Gallen International Breast Cancer Conference as follows (26): tumors with positive ER status, positive or negative PR status, no Her2 overexpression, and low Ki 67 were grouped into the “luminal A” group; those with ER positivity, PR positivity, and high Ki 67 or ER positivity and Her2 overexpression into the “luminal B” group; those with ER/PR- and Her2 positive phenotype into the “Her2” group; and tumors with neither hormone receptors nor Her2 amplification into the “triple negative” group.

Pathological response rates were assessed following surgical removal of tumors on hematoxylin and eosin stained slides. The pathological response to neoadjuvant chemotherapy was defined by using the Chevallier classification (I-IV) (class I – no residual carcinomas in breast or axillary nodes; class II – only in situ carcinoma remaining, nodes are negative; class III – invasive carcinoma with stromal fibrosis; and class IV – no or few modifications in the tumor), and class I and II is considered as pCR (27).

### Statistics

Statistical analyses were performed using Statistica 64 v12 (Statsoft Inc., Tulsa, OK, USA) and SPSS 15.0 Family Pack (SPSS, Inc., Chicago, IL, USA). For categorical variables, numbers were allocated for every investigated category. For continuous variables, the results are shown as means ± standard deviations, and median with interquartile range (IQR). Categorical variables were compared using χ^2^ test or Fisher exact method, depending on the number of the variables in the contingency tables. Disease-free and OS was estimated from the date of pathological diagnosis (core-biopsy sampling) to the date of last follow-up or death using the Kaplan-Meier survival probability estimator. Log-rank test was used to evaluate the effect of different variables on DFS and OS. All statistical tests were two-sided. Differences were considered to be statistically significant at *P* < 0.05.

## Results

We assessed the clinicopathological characteristics of the included 116 patients (Table 1). The patients’ median age at the time of diagnosis was 49.94 years (IQR 38-59). The median pretreatment tumor size assessed by PE was 40 mm (IQR 30-50) and if assessed by breast US it was median 27 mm (IQR 22-36 mm). According to the preoperative data the vast majority of patients had invasive ductal carcinoma (83.62%), while the others had invasive carcinoma not otherwise characterized, and lobular, mixed, and other types of carcinoma, each amounting to <10%. Most patients had T2 tumors (56.9%), 16.4% had T3, 12% had T4 tumors, and only 9.5% had T1 tumors.

**Table 1:** Pretreatment patient and tumor characteristics of 116 patients*

		n	%
Age	premenopausal	47	40.9
	perimenopausal	11	9.5
	postmenopausal	57	49.6
Clinical T stage	T1	11	9.5
	T2	66	56.9
	T3	19	16.4
	T4	14	12.1
	no data	6	5.1
Clinical N stage	node positive	67	57.8
	node negative	35	30.1
	no data	14	12.1
Histology	IDC	97	83.6
	ILC	3	2.6
	other	16	13.8
ER	positive	61	52.6
	negative	42	36.2
	no data	13	11.2
PR	positive	40	34.5
	negative	61	52.6
	no data	15	12.9
Her2/neu	positive	32	27.6
	negative	73	62.9
	no data	11	9.5
Neoadjuvant regimen	taxane	24	20.7
	anthracycline	21	18.1
	T+A	48	41.3
	trasuzumab	8	6.9
	other	15	13

*T – tumor, N– node, IDC– invasive ductal carcinoma, ILC – invasive lobular carcinoma, ER – estrogen receptor, PR – progesterone receptor, Her2 – human epidermal growth factor receptor 2, T+A– taxane + anthracycline.

Among 116 patients there were 67 node-positive cases (57.8%). With regard to hormone receptor status, 52.6% of the tumors were ER positive and 34.5% were PR positive.

Of 116 patients, 24 received taxane-based PST, 48 combined taxane + anthracycline treatment, 8 trastuzumab combinations, 21 anthracycline-based treatments, and 15 other treatments. In the taxane treated group, the pCR rate was 30%, in the taxane + anthracycline group 25%, in the anthracycline group 9.5%. and in trastuzumab group 37.5%.

Upon pathological review of tumor and nodal status, pathological complete or near-complete remission (pCR = Chevallier I and II) was observed in 25 of 116 cases (21.5%), 44% of whom had triple negative histology and 76% had high-grade tumor. According to the preoperative characteristics of the 25 tumors achieving pCR, 11 of the cases were triple negative, 7 were luminal B, and 7 were Her2 positive. Only 10 luminal-A patients were enrolled in this study, and all of these patients failed to achieve pCR. The same was true for the majority of luminal B tumors (35/42, 83.4%).

Univariate regression analysis was used to estimate the effects of clinical and pathological characteristics on response to neoadjuvant chemotherapy. Negative ER and PR status and Her2 positivity were the factors associated with an increased percentage of pCR (Table 2).

**Table 2:** Univariate predictors of pCR to neoadjuvant chemotherapy for breast cancer*

Characteristics	*P*
PR negativity	0.004
HER2 positivity	0.027
ER negativity	0.002
Therapy	NS

*pCR – pathological complete response, ER –estrogen receptor, PR – progesterone receptor, Her2 – human epidermal growth factor receptor 2, NS – not significant.

The menopausal status was not associated with the likelihood of achieving pCR. We did not find any significant correlation (Chi square: 4.76, df = 2, *P* = 0.093). But in the pCR group patients’ mean age was significantly lower than in the non-pCR group (44.4 ± 12.3 vs 50.8 ± 11.8, *P* = 0.017).

PE and US measurements were also compared with the residual pathologic tumor size. According to the PE data and UICC evaluation criteria, 27.6% of the patients achieved a clinical CR. However, the pathological complete response rate was lower: 21.5%. According to the results obtained by US, the clinical CR rate was 15.5% but we had the US measurement data for only 58 patients (Tables 3 and 4).

**Table 3:** The results of physical examination compared to pathological response after primary systemic therapy (n = 105 patients, unknown PE data in 11 cases)*

	PE - CR	PE - PR	PE - SD
Chevallier I+II	16 (55.2%)	9 (15.3%)	0 (0%)
Chevallier III	12 (41.4%)	38 (64.4%)	8 (47.1%)
Chevallier IV	1 (3.4%)	12 (20.3%)	9 (52.9%)
Total	29 (100%)	59(100%)	17 (100%)

*PE – physical examination, CR – complete response, PR – partial response, SD – stable disease.

**Table 4:** The results of breast ultrasonography compared to pathological response after primary systemic therapy (n = 58 patients, US restaging was incomplete in 58 patients)*

	US - CR	US - PR	US - SD	US - PD
Chevallier I+II	5 (55.6%)	9 (24.3%)	0 (0%)	0
Chevallier III	4 (44.4%)	25 (67.6%)	8 (72.7%)	0
Chevallier IV	0 (%)	3 (8.1%)	3 (27.3%)	1 (100%)
Total	9 (100%)	37 (100%)	11 (100%)	1 (100%)

*US – ultrasound, CR – complete response, PR – partial response, SD – stable disease, PD –progressive disease.

Of the 25 patients who achieved a complete pathological response, 9 were clinically described as partial clinical responders; the remaining were described as complete responders using PE. Based on the results of US for clinical evaluation of the 14 patients with available data from this group 5 achieved a complete pathological response and 9 achieved partial response (Tables 3 and 4). After neoadjuvant chemotherapy, both PE- and US-measured clinical remission associated significantly with pathological remission, (*P* < 0.001 and *P* = 0.004, respectively).

We further analyzed whether in pCR cases US added an additional value to PE evaluation. We found that in cases when PE correctly identified pCR, only 50% of US examinations showed complete remission – the false positivity rate was high. In those pCR cases when PE was false positive, only one US examination contradicted the result of PE by showing clinical complete remission. Thus, US did not add any additional diagnostic value to PE.

**Figure 1:**
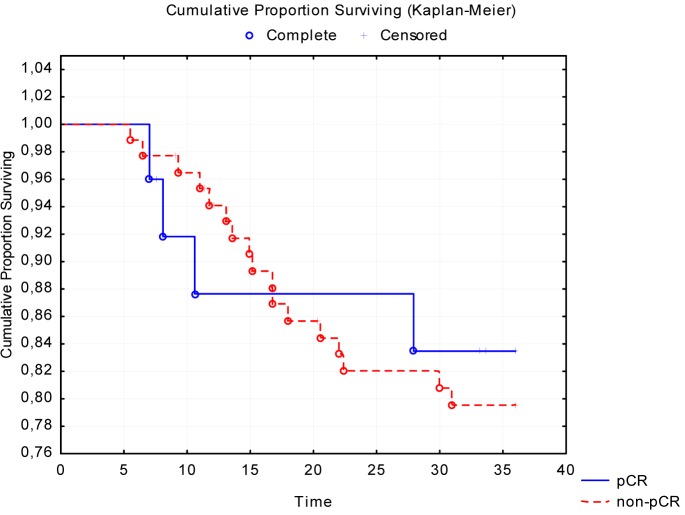
Disease free survival of pathological complete response (pCR) group compared to non pCR group, not significant *P* = 0.804.

**Figure 2:**
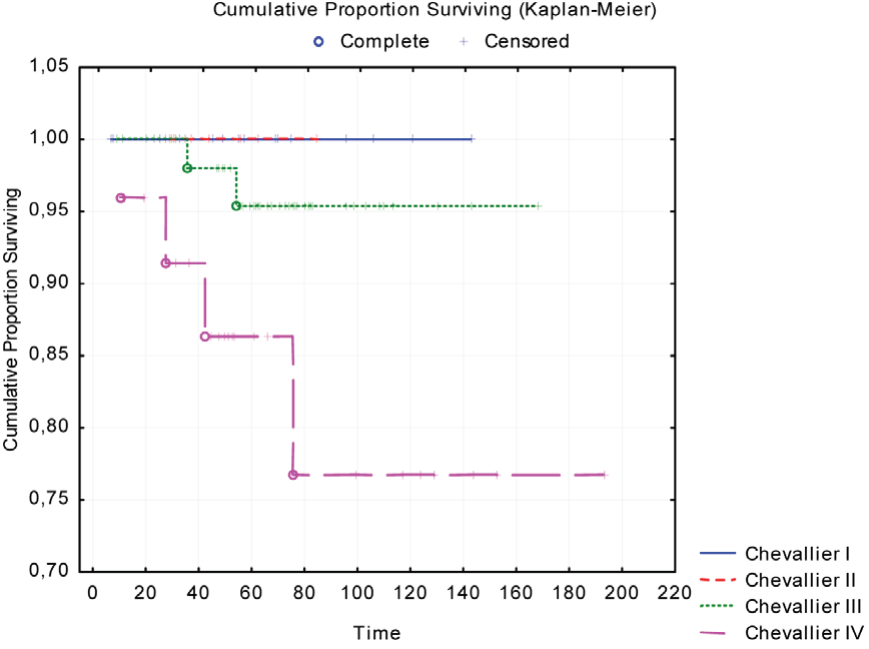
Overall survival in the four different Chevallier groups, not significant (*P* = 0.07). Significant differences we found between the Chevallier III and IV groups (*P* = 0.031).

The median follow-up was 56.1 months (IQR 36.3-77.1 months). Concerning DFS, pCR was not associated with better outcome (*P* = 0.804), however the number of patients with early disease progression in the pCR group was lower than in the non-pCR group (3 vs 15), but the difference was not significant (Figure 1). We also did not find significantly better OS in the pCR group (*P* = 0.237), but it should be noted that in the pCR group there were fewer events (CH I-II) during the follow-up period. Nonetheless, when we compared the four Chevallier subgroups regarding OS, we still not find differences (*P* = 0.079) however with subgroup analysis between the Chevallier III and IV groups we detected significant differences in the OS time (*P* = 0.031) (Figure 2).

## Discussion

In this study, we evaluated the accuracy of PE and US for clinical staging of primary breast cancer in women receiving PST, by correlating the results with pathologic measurement performed on surgical specimens and to determine the breast carcinoma subgroups likely to respond to PST.

The assessment of residual tumor size is important in planning the initial treatment course and also in monitoring disease response to the treatment. There are controversies regarding the reliability of the methods used to evaluate the size of residual breast carcinomas. Physical examination, US, mammography, and MRI have all been used to assess tumor size before, during, and after neoadjuvant chemotherapy in the everyday practice. A recent study by our team assessed the tumor response by our novel, breast cancer specific FDG-PET/CT criteria, which accurately differentiated pCR from non-pCR patients (28). However, the availability and costs of novel methods indicate that we should evaluate and investigate more classical techniques of tumor measurement. PE, US, and mammography are frequently used techniques for tumor measurements, but high false positivity rates (20%, 65%, and 46%, respectively) and notable false negativity rates (57%, 10%, and 20%, respectively) were published. Earlier studies suggested that PE was the best noninvasive predictor of the real size of breast cancer, but MRI can give the best correlation with pathology (12,29-31). When comparing the methods used for clinical assessment with final pathological findings the published results are heterogeneous, but still showing high correlation for PE and for US (32). We found that both PE and US were associated significantly with the final histology, however, PE showed slightly better results than US. The limitation of PE is that tumors smaller than 2 cm sometimes are not detectable. In contrast, if a large tumor shows considerable decrease in size by clinical examination, there could also be remaining small tumor foci with minimal residual disease. These small foci, scattered in a relative large area, could be defined as residual tumor or stable disease by the final pathological assessment. This result implies that the clinical diagnosis of cCR does not necessarily reflect the pathologic CR. It also means that the level of inaccuracy must be taken into consideration when assessing patient’s suitability for breast conserving surgery or for alternative chemotherapy. Even if cCR is achieved, it is possible that viable tumor tissue is still present at the primary site in some cases. It is generally accepted that three types of information can be used to estimate the probability of pCR: the tumor response after two courses of treatment, molecular markers, and clinical phenotype including hormone receptor status, tumor subtype, grade, and age (1,22). Several trials indicated that the absence of any response after the first two cycles was predictive for low probability of pCR even after completing chemotherapy (13,33). The majority of researchers agree that patients with ER negative and HER2-amplified breast cancer are more likely to achieve pCR (1,7,10,34,35). In our study, of the 25 tumors achieving pCR, 11 were triple negative, 7 were luminal B, and 7 were Her2 positive. This result is consistent with recently published data of other groups (4,7,19,36). Tan et al using a multivariate analysis have found that negative hormone receptor status, N0 nodal status before therapy, and HER2 amplifications are independent predictors of pCR (22).

The association between pCR and DFS or OS is always questionable. Large clinical trials of neoadjuvant therapy have demonstrated that patients with pCR have better DFS and OS compared with those with residual tumors (36,37). Fisher et al (38) concluded that long term DFS and OS were similar after neoadjuvant and adjuvant chemotherapies when similar chemotherapy regimens were used. We found that pCR was not associated with significantly better outcome, however, it should be highlighted that in the pCR group the number of early disease progression was significantly lower than in the non-pCR group (3 vs 15). Tan et al (22) by analyzing 518 breast cancer patients receiving neoadjuvant therapy also concluded that OS was not significantly different in patients with pCR and with residual disease. It needs to be mentioned that the follow-up period in the mentioned study (22) was rather short (<4 years). Similar results were reported by Jung et al (39) and the recently published meta-regression analysis by Berruti et al (40).

In conclusion, we found that both PE and US measured clinical remission was associated significantly with final pathology results, but PE was slightly more accurate than US. Serial US did not provide additional useful information in the majority of cases, but provided useful additional information in questionable cases. Imaging techniques like mammography, US, and MRI can help in cases when PE fails to identify the tumor. We determined the breast cancer subgroups likely to respond to primary systemic therapy and we found that patients with ER and PR negative, Her2-positive cancers were more likely to achieve pCR. Finally, pCR was not associated with significantly better DFS. Concerning OS, significant difference was observed between the Chevallier III and IV group and fewer events were observed in the pCR group.

The most important limitation of our study was the small number of events during the follow-up period therefore we were not able to analyze OS in different tumor subgroups comparatively. Additionally should be highlighted that the number of US examination was lower than expected due to strict inclusion criteria.

PE and US are the most generally used diagnostic methods worldwide in the prediction of residual tumor after neoadjuvant chemotherapy. We conclude that PE should be the basic method for evaluation of breast tumors during PST in classical candidates with locally advanced, T2 or larger, node positive tumors.
